# The Hepatocyte Expansion Paradox: A Review of In Vitro Challenges and Advances

**DOI:** 10.3390/cells15161465

**Published:** 2026-08-15

**Authors:** Mina Kolahdouzmohammadi, Nicholas Tjandra, Kevan Wu, Raha Nikoumaram, Graziano Oldani

**Affiliations:** 1 Department of Surgery, Faculty of Medicine, University of British Columbia, Vancouver, BC V6T 1Z3, Canada; 2 BC Children’s Hospital Research Institute, Vancouver, BC V5Z 4H4, Canada; 3 Faculty of Health Sciences, Simon Fraser University, Burnaby, BC V5A 1S6, Canada; nicholas.tjandra@bcchr.ca; 4 Faculty of Pharmaceutical Sciences, University of British Columbia, Vancouver, BC V6T 1Z3, Canada; 5 Faculty of Science, University of British Columbia, Vancouver, BC V6T 1Z3, Canada

**Keywords:** liver, primary hepatocytes, hepatocyte proliferation, in vitro culture, hepatic function maintenance

## Abstract

**Highlights:**

**What are the main findings?**

- Primary hepatocytes are notoriously difficult to maintain long-term in vitro, but defined growth-factor protocols can reversibly convert them into a proliferative, progenitor-like state before restoring maturity.
- Three-dimensional (3D) culture strategies improve hepatocyte maturation but remains limited in scalability.

**What are the implication of the main finding?**

- Integrating reprogramming, pathway modulation, and 3D-culture strategies is recommended to build a functionally stable hepatocyte platform.
- The translation of findings from animal studies to humans should be carefully considered before clinical application.

**Abstract:**

The adult liver exhibits remarkable regenerative capacity in vivo; however, primary hepatocytes (PHs), the principal functional cells of the liver, swiftly forfeit their proliferative potential and specialized hepatic functions when isolated from their native microenvironment and cultured in vitro. While PHs are the benchmark for studying hepatic physiology, xenobiotic metabolism, and toxicological responses, their rapid dedifferentiation resulting in the loss of hepatic functions significantly limits their further application. Recent studies suggest three converging strategies to address the challenge of long-term maintenance and expansion of PHs. First, defined chemical and growth factor-based protocols can temporarily induce hepatocytes into a proliferative, progenitor-like state, followed by a maturation phase that restores differentiated hepatic functions. In addition, the inhibition of signaling pathways linked to cellular stress responses and identity loss can postpone dedifferentiation and preserve drug-metabolizing activity for prolonged durations, thereby enhancing disease modeling and toxicology studies. Finally, three-dimensional (3D) culture platforms generally improve hepatocyte maturation and functional stability, but these are often not scalable due to matrix dependence, technical complexity, handling requirements, and cost. This review thoroughly examines innovative methodological advancements designed to facilitate the proliferation and prolonged viability of healthy PHs, focusing on their translational relevance and clinical applicability.

## 1. Introduction

The liver mainly consists of two epithelial cell types, namely hepatocytes and cholangiocytes, which derive from hepatoblasts throughout fetal development. Hepatocytes are often binucleated and manifest as sheets inside liver tissue [1]. In vivo, the adult liver has an impressive ability to regenerate, but primary hepatocytes (PHs), the gold standard for studying liver physiology and pharmacology, quickly lose their ability to proliferate and their specialized functions when isolated from their native environment [2]. Hepatocytes isolated from a donor liver show transient viability and functionality in culture, and usually decline within days due to loss of polarity, reduced expression of liver-specific genes, and cellular senescence [3,4]. This transition is characterized by a marked downregulation of hepatocyte-specific markers such as albumin and hepatocyte nuclear factor 4 alpha (HNF4A) and of genes involved in metabolism, in particular genes involved in Phase I xenobiotic metabolism. In addition, the genes controlling oxidative stress are diminished, which makes the in vitro hepatocytes more prone to oxidative injury than their in vivo counterparts [5].

Liver cancer and cirrhosis together cause more than two million deaths around the world every year. The fact that liver transplantation, the only known cure, fails to meet clinical demand exacerbates this problem [6]. Transplantation can only support a small fraction of patients with end-stage liver disease globally, and there is significant regional disparity in donor variability [7]. Therefore, hepatocyte transplantation has emerged as a promising therapeutic strategy [8]. However, its widespread clinical application remains constrained by the limited availability of sufficient numbers of functional hepatocytes. Additional challenges include difficulties in isolating viable cells from donor organs, poor cell survival following cryopreservation, low engraftment efficiency, limited post-transplant proliferation, and the persistent risk of allograft rejection [6,9].

**Figure 1:**
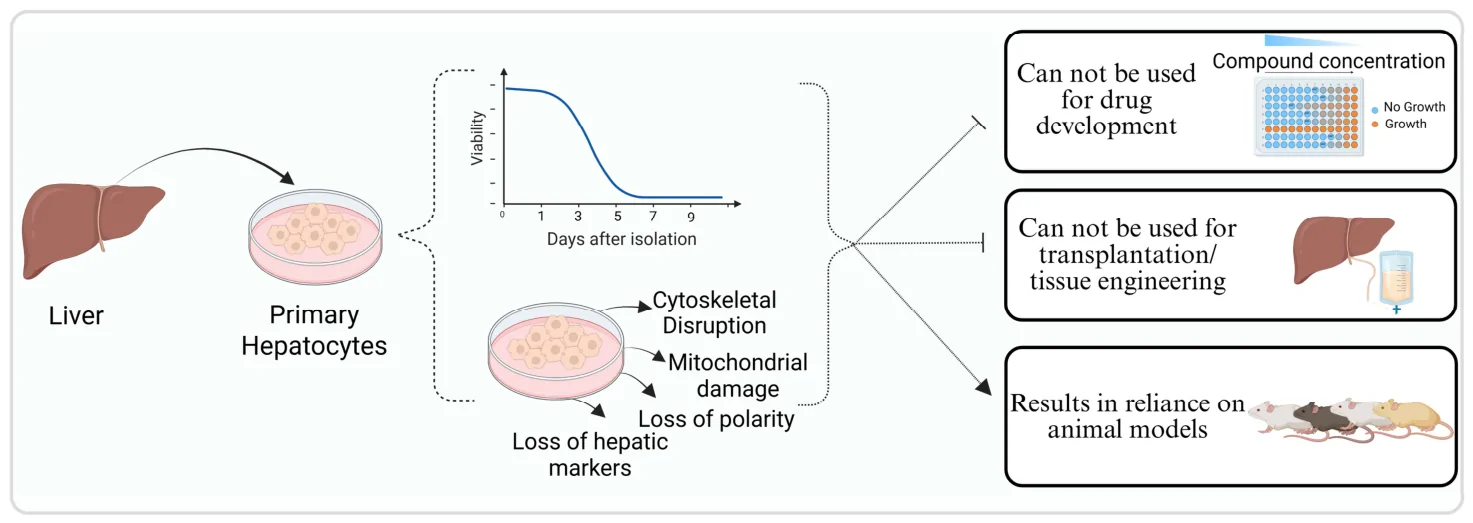
PHs’ challenges after isolation from their natural tissue environment. After isolation, PHs demonstrate a gradual decline in viability, characterized by cytoskeletal disruption, mitochondrial damage, loss of cellular polarity, and downregulation of hepatocyte-specific markers. These modifications significantly restrict their application in drug discovery and transplantation or tissue engineering, hence mandating ongoing dependence on animal models for hepatic research. Created in BioRender. Kolahdouzmohammadi, M. (2026) https://BioRender.com/yj80u63 (accessed on 12 August 2026).

Thus, maintenance and expansion of mature, functional hepatocytes in vitro would have a major effect on scalable cell-based therapies, disease modeling, reduction in animal studies, and acceleration of drug development procedures [10] (Figure 1).

This review critically examines the key obstacles limiting hepatocyte expansion and surveys the methodological breakthroughs of the past decade that have meaningfully advanced the long-term culture and functional maintenance of healthy PHs.

## 2. Challenges in PH Expansion

A primary obstacle to PH proliferation is their final differentiation and quiescent state. In an adult liver, hepatocytes mostly exist in the G0 phase of the cell cycle throughout homeostasis [11]. After partial hepatectomy or injury, hepatocytes can re-enter the cell cycle and proliferate extensively in vivo, but this regenerative response requires a tightly controlled interplay of mitogenic growth factors, cytokines, metabolic cues, and mechanical stimuli that are difficult to recapitulate ex vivo [12]. PHs undergo severe morphological and functional deterioration within 24–48 h in two-dimensional (2D) monolayer culture, characterized by a loss of polygonal shape, disruption of tight junctions, collapse of bile canaliculi, and gradual downregulation of liver-specific genes [13]. These alterations eventually result in cellular senescence and severely restrict long-term culture viability.

The failure to recapitulate the complexity of the hepatic environment is a major contributor to hepatocyte dedifferentiation. In vivo, hepatocytes are situated in a highly organized lobular architecture and dynamically interact with a complex extracellular matrix (ECM) of collagens, laminins and fibronectin [14]. This ECM provides important biochemical and biomechanical cues to modulate polarity, survival, and proliferation. When hepatocytes are maintained in vitro, however, this environment is simplified by using conventional culture substrates, leading to dysregulated integrin signaling and accelerated functional decline [14].

The absence of interactions among non-parenchymal cells (NPCs) should also not be overlooked. Hepatocytes in the liver consistently engage with sinusoidal endothelial cells, hepatic stellate cells, and Kupffer cells, together regulating metabolic zonation, regenerative signaling, and immunological homeostasis. The absence of paracrine and juxtacrine connections in monoculture systems exacerbates hepatocyte survival and growth potential [14].

PHs exhibit considerable inter-donor variation in proliferative capacity, drug metabolism and stress tolerance, which are influenced by donor age, genetic background, clinical status and drug exposure history [15]. Such variability makes experimental standards difficult and limits scalability. Hepatocytes derived from animals are more accessible; however, the presence of species-specific metabolic pathways and regulatory networks significantly limits their translational value.

Collectively, these challenges highlight the need for advanced culture procedures that more closely recapitulate the native hepatic niche.

## 3. Recent Strategies for Hepatocyte Expansion

**Figure 2:**
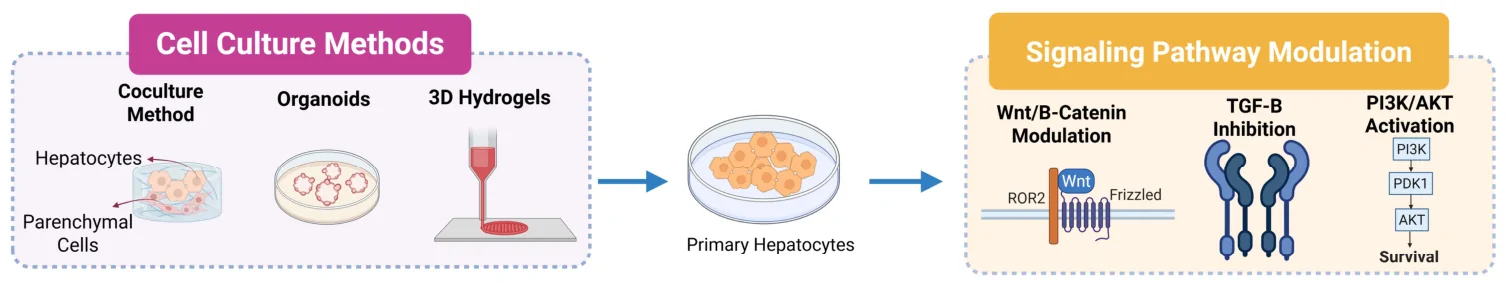
Recent Strategies for PHs Expansion in vitro. Schematic overview of major approaches used to enhance PHs proliferation and maintenance, including cell culture methods (co-culture with parenchymal/supporting cells, organoid-based systems, and 3D hydrogel platforms) and signaling pathway modulation to improve survival and expansion capacity, such as Wnt/β-catenin activation, TGF-β inhibition, and PI3K/AKT activation. Created in BioRender. Kolahdouzmohammadi, M. (2026) https://BioRender.com/yj80u63 (accessed on 12 August 2026).

Current PHs expansion utilizes several tactics, such as using a cocktail of small molecules (SMs), augmentation of the ECM, and the application of 3D culture methodologies. All of these techniques may operate synergistically or can be utilized as separate methodologies, with the main aim of preserving functional PHs for a longer period (Figure 2). However, these approaches differ in whether they achieve a genuine increase in hepatocyte number or simply extend the functional lifespan of an existing cell population. Throughout this review, we therefore tried to distinguish studies demonstrating a quantifiable increase in cell number (via passage number or fold-expansion), marked as true expansion (TE), from those reporting extended viability or function without a corresponding increase in cell number, marked as functional maintenance (FM).

### 3.1. Chemical Modulation of Hepatocyte Culture Media

**Figure 3:**
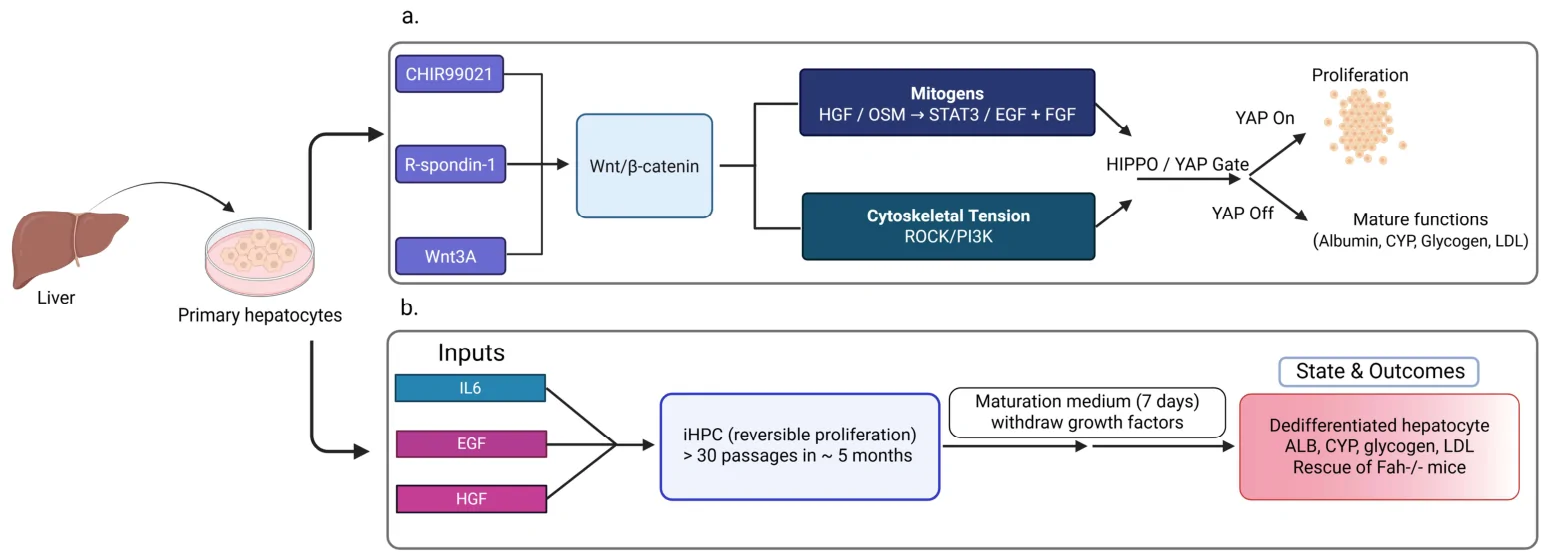
(**a**) Wnt/β-catenin-centered reversible expansion framework. Small-molecule or ligand inputs (CHIR99021, Wnt3A, and R-spondin-1) activate Wnt/β-catenin, while mitogens (HGF, OSM activating STAT3, and EGF plus FGF) and cytoskeletal tension pathways (ROCK and PI3K) feed into a Hippo/YAP gate that determines state. YAP on favors proliferation; YAP off favors recovery of mature hepatocyte functions, including albumin secretion, CYP activity, glycogen storage, and LDL uptake. (**b**) Cytokine and growth factor summation to a reversible iHPC state. IL-6 priming is combined with EGF or HGF to sum STAT3, ERK, and PI3K/AKT signals, converting PHs to induced hepatic progenitor cells that can be passaged for more than 30 passages over about five months with passaging every 5–7 days at near confluence [18]. Switching to a defined maturation medium for seven days and withdrawing growth signals restores hepatocyte functions, and transplanted cells rescue Fah-/- mice, consistent with the requirement for co-stimulation to preserve redifferentiation capacity. Created in BioRender. Kolahdouzmohammadi, M. (2026) https://BioRender.com/yj80u63 (accessed on 12 August 2026).

Recent advancements have transformed difficult-to-cultivate PHs into expandable, transplantable cells. A consistent protocol is observed in both 3D organoids and simpler 2D formats: stimulate Wnt/β-catenin (typically through Wnt3a and/or R-spondin) [16], augment with conventional mitogens, such as hepatocyte growth factor (HGF) and epidermal growth factor (EGF), and occasionally incorporate Oncostatin M (OSM) [17] (Figure 3a). These signals initiate a regeneration-like phase: quiescent hepatocytes re-enter the cell cycle and experience significant expansion while maintaining the ability to regain a mature hepatocyte phenotype upon the cessation of mitogenic stimuli [10]. The dynamics of this reversible state are most visible in vivo. Hu et al. demonstrated that hepatocyte-derived organoids undergo an initial 30-day quiescent phase post-transplantation, succeeded by rapid and extensive liver repopulation akin to that of PHs [16].

To maintain PHs identity during prolonged culture, Igarashi et al. systematically identified essential niche-derived signaling inputs [17]. Their protocol incorporated β-catenin stimulation (Wnt3A or R-spondin), survival and motility signals (EGF or HGF), epithelial stimulation (fibroblast growth factor 10 (FGF10)), STAT3 activation (interleukin-6 (IL-6) or OSM), protein kinase A (PKA) signaling (forskolin), and transforming growth factor beta (TGF-β) inhibition (A83-01 or Noggin). Under these specific conditions, mature identity—assessed via albumin secretion, cytochrome P450 (CYP450) activity, urea synthesis, bile canalicular organization, and functional liver repopulation following xenotransplantation—was maintained for several months, with reported expansion approaching 10^6^-fold over ~4 weeks, exceeding the expansion and functional benchmarks reported in earlier hepatocyte organoid systems, supporting classification as TE [17]. This system, while robust, is limited by its dependence on Matrigel and the complexity of its multi-factor cytokine composition, which together constrain scalability and cost-effectiveness for large-scale or clinical application.

In addition to extensive niche replacement, other cytokine-driven approaches have shown that reversible hepatocyte expansion can occur independently of canonical Wnt signaling. The synchronized stimulation of IL-6, EGF, and HGF stimulates STAT3, ERK, and PI3K/AKT signaling pathways, facilitating the transformation of adult hepatocytes into induced hepatic progenitor cells (iHPCs) [18]. These iHPCs proliferate for approximately 30 passages and thereafter redifferentiate to restore albumin secretion, CYP450 activity, glycogen storage, and low-density lipoprotein (LDL) uptake, while also rescuing Fah^−^/^−^ mice following transplantation, supporting classification as TE. Dose–response studies indicate that IL-6 initiates proliferation, but elevated IL-6 in conjunction with EGF/HGF maintains prolonged cycling. Single-cell multi-omics identifies ERK, PI3K, and Src activation as early dedifferentiation markers and uncovers initial TGF-β signatures, suggesting a potential role for TGF-β modulation in stabilizing identity during expansion [18] (Figure 3b).

Approaches that reduce system complexity have resulted in minimalist chemical methodologies that preserve reversibility. Jiang et al. employed ROCK inhibitor Y-27632 and GSK3β inhibitor CHIR99021 to reduce actomyosin tension and stabilize β-catenin, therefore creating a proliferative but reversible progenitor-like state [19]. Following withdrawal into hepatic maturation media, the expression of albumin and HNF4A was restored, although levels of alpha-fetoprotein (AFP) and Ki-67 diminished. A combination of the same two chemicals augmented early liver repopulation post-transplantation; however, the long-term sustainability of this effect remains debatable [19].

Notably, when expansion is no longer the objective and the maintenance of mature hepatocyte function is prioritized, biochemical signaling is subordinated to mechanical and cytoskeletal regulation. Sun et al. have demonstrated that inhibiting cell spreading and reducing actin tension, Src kinase, tankyrase and PI3K all inhibit Yes-associated protein (YAP) activation. This inhibition enables hepatocytes to preserve polarity and metabolic activity without re-entering the cell cycle [20]. Under mechanically specified circumstances, mature hepatocyte function can be sustained for around two weeks in certain matrix-independent culture platforms. In addition, the ECM significantly modulates hepatocyte plasticity. Collagen, laminin, Matrigel, and decellularized liver scaffolds enhance hepatic identity; however, they reduce actomyosin tension, which is achieved through ROCK inhibition, micropatterning, or tension-modulating cocktails, increasing the risk of dedifferentiation. Expansion is generally facilitated by diploid (2c) hepatocytes, and the interaction between Wnt and Hippo/YAP signaling indicates that mechanically induced YAP activation can either promote regulated plasticity or accelerate identity loss, depending on the culture context [20].

At the most basic regulatory level, epigenetic control is crucial for hepatocyte plasticity. The activity of SIRT1 is essential for the transformation of PHs into proliferative progenitor-like cells; the inhibition of SIRT1 results in growth cessation and triggers apoptosis [10]. The use of valproic acid induces chromatin relaxation in PHs, facilitating long-term, karyotypically stable development, effective gene modification, and therapeutic restoration following microencapsulated transplantation [21]. Table 1 presents a summary of studies from the current decade that utilize pharmacological changes to develop viable, expandable hepatocytes.

### 3.2. Co-Culture and 3D Systems as a Platform for Having Functional PHs in Long Term

Conventional 2D cell culture systems have been acknowledged as insufficient for the sustained preservation of PHs differentiation, polarity, and metabolic function [24,25]. Across multiple studies, hepatocytes cultured in 2D systems swiftly impair liver-specific gene expression, CYP450 activity, and synthetic capability, ultimately leading to reduced viability and inadequate predictive accuracy for drug testing [24,25]. These limitations are mainly attributed to the absence of a physiologically relevant 3D microenvironment that is needed to preserve the structural and metabolic signals of the native liver tissue.

There is growing evidence that hepatocyte physiology is tightly regulated by cell-ECM cross-talk in a 3D architectural context [17]. Consequently, a wide variety of complex culture platforms have been developed to better mimic the hepatic niche. These include sandwich cultures, scaffold-based systems, bioreactors, and microfluidic liver-on-a-chip technologies [4,5,10]. Although these systems have shown to be consistently superior to conventional monolayer cultures in terms of hepatocyte functionality and viability, they suffer from intrinsic limitations. Often, the problems cited are poor scalability, non-specific adsorption of SMs onto scaffold materials, technical complexity, and the large batch-to-batch variability of ECM components, impairing reproducibility [21].

Within 3D methodologies, hepatocyte spheroids and organoid-like aggregates emerged as notably robust and reproducible models [19,20,25,26]. These self-assembled microtissues facilitate improved cell–cell interactions and have greater physiological relevance in cell polarity than 2D systems [19,20,25,26]. Multiple comparative studies indicate that spheroid cultures sustain elevated liver-specific functions, such as albumin secretion, urea production, and drug-metabolizing enzyme activity, while simultaneously demonstrating enhanced sensitivity to hepatotoxic compounds and a longer lifespan [26,27]. Spheroids significantly promote the development of bile canaliculi-like networks and maintain stable metabolic zonation, both of which are characteristics that are infrequently preserved in 2D environments [19].

Scaffold-based 3D models emphasize the importance of recapitulating the biochemical and biophysical characteristics of the hepatic environment. Porous polymeric scaffolds composed of poly (lactic-co-glycolic acid) (PLGA) and modified with a coating of ECM components such as collagen have greatly enhanced hepatocyte adhesion and function [28]. Electrospun scaffolds with pore sizes and structures similar to native liver tissue often outperform non-porous or unmodified materials, permitting additional albumin secretion, urea synthesis, and CYP450 activity [28]. In addition to structural support, scaffold stiffness, degradability, and proteolytic stability are all important factors affecting hepatocyte behavior, emphasizing the importance of closely mimicking the biomechanical properties of liver tissue in the design of in vitro models [29].

Despite these advances, there is increasing evidence that 3D environment alone is not sufficient to fully preserve hepatocyte phenotype. The importance of incorporating optimal ECM-derived biochemical cues in 3D systems has been demonstrated in numerous studies. Self-assembling peptide hydrogels formed from Fmoc-FF alone failed to maintain hepatocyte viability or function, but the addition of integrin-binding motifs such as RGD (Arg-Gly-Asp) dramatically increased cell survival and CYP450 activity, surpassing both 2D cultures and Matrigel for long-term cultures [30]. Moreover, the combination of inflammatory and regenerative signals can prolong the in vitro lifespan of PHs. Compact 3D liver models enhanced with tumor necrosis factor-α (TNFα) have demonstrated the capacity to sustain long-term hepatocyte culture, encompassing serial passaging and expansion over several months, and thus illustrate the importance of precisely regulated inflammatory signaling in facilitating hepatocyte plasticity and viability [31]. These approaches cast inflammation as a modifiable element of the in vitro hepatic niche and challenge the conventional wisdom that it only has detrimental effects on cellular longevity.

Co-culture techniques provide an additional layer of complexity that further improves hepatocyte stability by re-establishing intercellular signaling pathways present in native liver tissue. In this regard, small hepatocyte progenitor-like cells (SHPCs) were derived from adult PHHs by direct co-culture with irradiated mouse embryonic fibroblasts (MEFs), which provided feeder-dependent signals that triggered proliferation while maintaining necessary hepatocyte features [32]. Under optimized conditions, PHH-derived SHPCs exhibited polarized morphology, functional bile canaliculi, persistent expression of hepatocyte-specific transcription factors, and transporters and maintained CYP450 activity, leading to drug metabolism capacity comparable to that of PHs. These features underscored their potential significance [32].

The literature collectively shows no singular 3D culture method is adequate to entirely replicate PHs function independently. The most effective systems incorporate 3D architecture, customized ECM composition, biomechanical signals, inflammatory pathways, and multicellular interactions. These convergent design concepts are progressively influencing the development of next-generation liver models for drug discovery, disease modeling, and regenerative medicine, as outlined in Table 2.

### 3.3. Hepatocyte Expansion Through Transcriptional Reinforcement and Optimized Isolation

Alternatively, hepatocyte proliferation could be enhanced by reinforcing hepatocellular identity through specific transcriptional regulation [37], rather than microenvironmental signals alone. In this regard, the liver-enriched transcription factors FoxA3 and Hnf4α have been utilized based on their complementary and indispensable roles in hepatocyte biology. FoxA3 is a pioneer transcription factor that opens condensed chromatin for access to hepatic gene regulatory networks, promoting lineage competence and regenerative capacity. Hnf4α acts in parallel as a master regulator of hepatocyte differentiation and metabolic gene expression, thus reinforcing mature hepatic functions while suppressing dedifferentiation. Co-overexpression of FoxA3 and Hnf4α in PHs has been reported to maintain proliferation, while preserving key hepatocyte-specific features, such as expression of mature hepatic markers, glycogen storage ability, and functional indocyanine green transport [37].

In addition, advances in PHs isolation techniques improve the availability of functional hepatocytes, thereby alleviating a significant upstream bottleneck for expansion platforms. A novel isolation procedure has been established to retrieve functional PHs from tiny, non-encapsulated liver resection surplus that is inappropriate for standard perfusion methods. Green et al. integrated mechanical tissue dissociation with sequential ethylene glycol tetraacetic acid (EGTA) and collagenase digestion, along with red blood cell lysis to reduce contamination and cytotoxicity, resulting in viable hepatocytes that maintain essential hepatic functions, such as albumin and CYP450 expression and urea secretion (38). This approach ensures hepatocyte viability is predominantly unaffected by donor steatosis and other confounding tissue characteristics, facilitating the wider use of clinical specimens as a source of expansion-competent cells (Table 3).

## 4. Conclusions

Hepatocytes are widely used in basic research and clinical applications, including investigations of drug metabolism and toxicity as well as therapeutic use in patients with acute liver failure and terminal liver diseases. However, the limited proliferative capacity of PHs, often associated with reduced viability and progressive loss of hepatocyte-specific functions in vitro, has constrained their broader contribution to health and science [39].

In the recent decade, this paradigm has undergone significant transformation. Different strategies have been implemented to keep PHs functional and mature in vitro. Among the most impactful advances has been the rise of 3D liver organoid systems, which better preserve cell identity and enable longer-term propagation compared with traditional 2D models while also supporting patient-specific disease modeling and drug testing approaches [40,41].

At the same time, the field has become more conceptually mature: rather than chasing a single “perfect hepatocyte,” researchers now use a toolbox of approaches including adult-derived organoids, chemically driven progenitor-like expansion, and PSC-derived hepatic systems [40,41,42]. Yet the core challenge remains unsolved: expanded hepatocytes often drift away from a stable adult state. PSC-derived hepatocyte-like cells illustrate this well; they generally show lower functional recapitulation than PHs, and most fail to self-renew post-differentiation, requiring repeated rounds of de novo differentiation that constrain their direct substitutability with adult hepatocyte systems [40]. More broadly, many expanded systems still display immature or mixed hepatic phenotypes, variability across donors and protocols, and incomplete recovery of key adult functions such as robust CYP activity and long-term metabolic competence. In other words, we are increasingly good at making “liver-like cells” but less consistent at maintaining adult-like hepatocytes that behave predictably across time and laboratories.

Caution is warranted when extrapolating hepatocyte expansion findings from mouse to human systems. Several of the most dramatic expansion results reported to date—including near-unlimited theoretical fold-expansion and multi-month clonal proliferation—have been demonstrated almost exclusively in mouse hepatocytes [18,31], whereas human systems have generally achieved more modest expansion with measurable trade-offs in redifferentiation and repopulation capacity at higher expansion levels [4,17]. Human primary hepatocytes also show substantial donor-to-donor variability that is not typically observed to the same degree in inbred mouse models, and this variability can persist even within engineered expansion or maturation protocols derived from the same donor [4,15]. Together, these differences suggest that expansion efficiency, functional recovery, and regenerative capacity established in mouse systems should not be assumed to translate directly to human hepatocytes, and findings from mouse models should be validated independently in human systems before conclusions are extended across species.

Moving forward, the next stage of progress will likely depend less on single-factor breakthroughs and more on standardization and integration. This includes (i) harmonized benchmarks for defining functional hepatocyte identity, (ii) scalable manufacturing and cryopreservation workflows, and (iii) improved microenvironment engineering with stromal support, biomaterials, and vascularization strategies. Together, these advances hold significant promise for future clinical translation, particularly for metabolic liver disorders such as Crigler-Najjar syndrome, ornithine transcarbamylase (OTC) deficiency, and urea cycle disorders where improved maintenance of functional hepatocytes would directly enhance engraftment outcomes.

Two important considerations remain as this field moves toward clinical translation. First, continued attention to genomic stability and tumorigenic risk is essential during long-term expansion of PHs, particularly for protocols involving sustained Wnt/YAP activation, extensive serial passaging, epigenetic modulation, or lentiviral overexpression of transcription factors such as Foxa3 and Hnf4a [17,37]. Second, caution is warranted when extrapolating hepatocyte expansion findings from mouse to human systems. PHHs show substantial donor-to-donor variability that is not typically observed to the same degree in inbred mouse models [15]. Findings from mouse models should therefore be validated independently in human systems before conclusions are extended across species.

Ultimately, hepatocyte expansion research is entering a productive “engineering phase,” where the question is no longer whether hepatocytes can be expanded, but whether we can expand them safely, reproducibly, and at adult-level function. Achieving that goal would not only transform liver disease modeling and drug development but also serve as a foundation for future regenerative therapies targeting liver failure and chronic liver disease.

## Figures and Tables

**Table 1:** Summary of studies using pharmacological/SM modifications to maintain viable, expandable hepatocytes.

Ref.	Targeted Signaling Pathway	Study Model	Number of Passages (TE or FM)	Time Point Study (Y/N)	Findings	Limitations	Additional Notes
Hu et al., 2018 [16]	Wnt pathway	In vitro: 3D Matrigel culture (PMHs, PHHs) In vivo: Fah^−/−^ NOD Rag1^−/−^ Il2rgnull (FNRG) mouse transplantation	>20P (mouse), up to 28P (human fetal) **TE**	Y	- Organoids stably expanded over 20+ passages with preserved morphology - Successfully engrafted and restored liver function in FNRG mice	Needs further verifications	R-spondin1-conditioned medium derived from 293T cells was used as Wnt agonist
Zhang et al. 2018 [4]	Wnt, YAP pathway	In vitro: PHHs (2D/3D) In vivo: FNRG mouse transplantation	>6P **TE**	Y	- HM expands PHHs ~10,000-fold while maintaining hybrid hepatocyte–progenitor phenotype. - Transplanted ProliHHs engraft efficiently and repopulate (~64%) FNRG mouse livers.	Donor variability noted across samples	- Bi-phenotypic “intermediate” status recapitulates natural liver regeneration. - Study supports disease modeling and drug screening.
Unzu et al. 2019 [22]	Wnt pathway	In vitro: 2D/3D PEG-based microwell PHHs In vivo: NOD-NSG mouse transplantation	>4P **TE**	Y	- Maintained genomic stability and classic hepatocyte markers -Redifferentiated cells regained metabolic function and supported HBV/HDV replication, with improved efficiency in 3D culture	-Limited in vivo engraftment or functionality - Long-term genomic and functional assessments post-expansion/redifferentiation not fully explored.	HPCs more similar to PHHs than iPSC-HLCs based on transcriptomics
Sun et al. 2019 [20]	Hippo/YAP signaling pathway	In vitro: 2D Matrigel (PMHs, PHHs) In vivo: Fah^−^/^−^ mouse transplantation	NA **FM**	Y	-Mechanical tension triggers hepatocyte dedifferentiation via YAP -LBDXL cocktail or confined spreading sustains hepatocyte functions and enables in vivo repopulation	- LBDXL hepatocytes failed to repopulate the livers of Fah^−^/^−^ mice at 4 weeks and later time points.	- LBDXL medium does not require Matrigel, but adhesion issues arise after 2 weeks
Fu et al. 2019 [10]	SIRT1-dependent deacetylase signaling	In vitro: PHHs (2D/3D) In vivo: Fah^−^/^−^ Rag2^−^/^−^ mouse transplantation	>10P **TE**	Y	- HepLPCs sustained expansion to P10 with stable karyotypes	- Long-term genomic stability after P10 not assessed.	-By P10 karyotype showed 2/3 lines diploid, and 1/3 with partial triploidy at Chr5 -Used lineage-tracing GFP-puro vector under TBG promoter to confirm hepatocyte origin
Chen et al., 2021 [5]	GSK-3α/β, ROCK, and TGF-β	In vitro: PHHs (2D)	NA **FM**	Y	Cells could be maintained for as long as 2 months and displayed excellent cell bioactivity	No in vivo validation	Suggests a closer transcriptomic resemblance to native liver tissue
Guo et al., 2022 [18]	JAK/STAT3, MAPK/ERK1/2, PI3K/AKT pathways	In vitro: PMHs (2D) In vivo: Fah^−^/^−^ mouse transplantation	>30P **TE**	Y	- IL6 + EGF + HGF enable long-term hepatocyte expansion & maintain differentiation capacity. - Successful liver repopulation in Fah^−/−^ mice	Needs validation on PHHs	IL-6 alone is inadequate; a combination with EGF/HGF is necessary
Jiang et al., 2023 [19]	ROCK and WNT/β-catenin pathway	In vitro: PMHs (2D) In vivo: Fah^−^/^−^ mouse transplantation	>30 P **TE**	Y	- YC (Y-27632 + CHIR99021) induced hepatocyte dedifferentiation into hepatic progenitor cells - Clinically used drugs NL (Netarsudil and LY2090314) also showed similar effects	Long-term safety in vivo was not fully assessed	NL suggested for clinical translation as alternative to research-grade YC combo
Li et al., 2024 [21]	Epigenetic regulation via HDAC1	In vitro: PPHs (2D) In vivo: C57BL/6J mouse transplantation	>20 P **TE**	Y	valproic acid promotes long-term in vitro expansion of PPHs and improved mouse survival in liver failure model	Needs more precise molecular mechanisms investigation	valproic acid-iHPCs maintain karyotype stability and progenitor marker expression for over 20 passages
Hao et al., 2025 [23]	ERK/MAPK, PI3K, Src and TGF-β	In vitro: PPHs (2D) In vivo: FRGN mouse transplantation	NA **FM**	Y	Identified key signaling pathways involved in hepatocyte dedifferentiation	Long-term safety and efficacy of the chemical combination require further investigation	Utilized 10× Genomics multiome technology for simultaneous single-cell RNA-seq and ATAC-seq
Igarashi et al., 2025 [17]	Wnt/β-catenin, STAT3, and YAP	In vitro: 3D PHHs In vivo: TK-NOG mice (xenotransplant)	Long-term passaging approximately every 2 weeks for over 3 months **TE**	Y	Combined activation of Wnt and STAT3 (via OSM) enables long-term self-renewal of adult human hepatocyte organoids while preserving hepatic identity	Scalability to human clinical scale remains to be addressed	- Million-fold expansion in ~4 weeks - Modeled MASLD and OTC deficiency

Hepatitis B Virus (HBV), Hepatitis D Virus (HDV), Hepatocyte Medium (HM), Hepatocyte-derived progenitor-like cells (HepLPCs), Hepatocyte-like cells (HLCs), Human hepatic progenitor cells (HPCs), induced Pluripotent Stem Cell (iPSC), Latrunculin B, Blebbistatin, Dasatinib, XAV939, LY294002 (LBDXL), Metabolic dysfunction-associated steatotic liver disease (MASLD), NOD/SCID/IL2Rγnull (NOG), Nonobese diabetic (NOD)-Cg-Prkdcscid Il2rgtm1Wjl/SzJ (NSG), Ornithine transcarbamylase (OTC), Polyethylene glycol (PEG), Primary human hepatocytes (PHHs), Primary mouse hepatocytes (PMHs), Primary pig hepatocytes (PPHs), Thymidine kinase (TK), Thyroxine-binding globulin (TBG), Valproic Acid (VPA).

**Table 2:** Summary of studies that used 3D and co-culture-based techniques to maintain and expand hepatocytes.

Ref.	Study Model	Time Point Study (Y/N)	Findings	Limitations	Additional Notes
Bell et al., 2016 [26]	In vitro: PHHs, 3D spheroids in chemically defined, serum-free conditions (ultra-low attachment plates)	Yes: assessed at 7 days post-aggregation and during time course up to 35 days	Three-dimensional spheroids closely match in vivo liver proteome, including inter-donor variability. Proteomes stable over 5 weeks vs. rapid deterioration in 2D.	No expansion	• Scalable and automatable spheroid formation. • Versatile: supports co-culture, disease modeling, chronic toxicity assays
Chang et al., 2017 [25]	In vitro: Liver-on-chip MPS vs. 2D monolayer culture	Yes: monitored viability up to 28 days and function to 14~15 days	MPS maintained higher viability (~>14 days) vs 2D (5–7 days)	No long-term culture beyond ~2 weeks; Focused on acute toxicity only (AFB_1_); broader chemical panels not tested; Cost and complexity of MPS systems vs. standard 2D cultures	Includes cross-species comparison (rat vs. human cells) in the same platform
Brown et al., 2018 [28]	In vitro: PHHs, Culture within Wet Electrospun PLGA-ECM Scaffolds	Yes: protein and synthetic function recorded over the course of 14 days	PLGA-collagen at 100 μg/mL improved albumin, urea, and CYP450 vs. unmodified PLGA; better than fibronectin scaffolds and sandwich control	The decline in CYP450 activity persisted in PHs; ECM component composition requires further optimization.	Scaffold pores optimized using wet electrospinning and resemble porous structures in the matrix of normal liver tissue
Garnier et al., 2018 [11]	In vitro: PHHs, 3D organoid culture on Matrigel	Yes: organoid number monitored at day 7 and 14. Cell number per well recorded from day ~20 to day 40. Mature hepatocyte markers observed every 24 h from 24 to 72 h after plating.	• 3D organoid culture induces proliferation and progenitor-like gene expression (Ki67, EpCAM, CK19, Sox9), with loss of mature markers (Albumin, CYP3A4). • Suspension format retains more mature marker expression (higher Albumin, HNF4α). • Growth peaked ~30 days and cells survived for more than 2 months prior depletion.	Substantial donor variability; Organoid cultures exhausted by ~2 months; No transcriptome-wide profiling performed.	• Suspension culture format is scalable and compatible with automation/bioreactors. • Demonstrates potential for large-scale hepatocyte production from cryopreserved sources.
Peng et al., 2018 [31]	In vitro: PMHs, 3D culture on Matrigel In vivo: Fah^−^/^−^ mouse transplantation	Yes: over 6 months. Albumin secretion and CYP3A11 activity monitored at 3, 5, and 7 months.	• TNFα significantly enhances hepatocyte colony formation and expansion in 3D culture. • Cells retain hepatocyte identity with broad marker expression. • Expanded hepatocytes successfully engraft and repopulate injured Fah–/– mouse livers.	-	• Demonstrates for the first time that inflammatory signals can sustain long-term culture of primary hepatocytes. • Combines cytokine and Wnt-based cues to mimic liver injury/regeneration niche.
Bell et al., 2018 [27]	In vitro: PHHs, 2D sandwich and 3D spheroid cultures	Yes: viability measured at 72 h, day 7, and day 14. Acetaminophen and dextrorphan formation measured over 14-day period.	Three-dimensional spheroids had higher functional stability and more sensitivity compared to 2D cultures of the same donors.	In vitro only, no in vivo validation; Focused on toxicity readouts; did not investigate molecular mechanisms; Donor-to-donor variability, though multicenter design partially addressed this.	• Highlighted multicenter reproducibility. • Supports adoption of 3D spheroid platforms in preclinical safety assessment.
Rose et al., 2021 [33]	In vitro: 3D collagen culture	Yes: Viability assessed over 28 days. Proliferation observed over 15 days in two waves: days 3–7 (wave one), days 8–13 (wave two).	• PHs proliferated in two distinct waves within the first 2 weeks. • Proliferative cells retained mature hepatocyte markers and high detoxification capacity. • Collagen-based spheroids maintained polarity and function for at least 28 days; MEK inhibition induced additional cell cycle entry.	No long-term expansion or passaging beyond early growth; Donor variability and scalability not deeply explored.	• 3D “Hepoid” system established, relying on stiffness and aggregation conditions. •Transient suppression of the MEK1/2–ERK1/2 (MAPK) pathway.
MacPherson et al., 2021 [30]	In vitro: PHHs co-cultured with J2 fibroblasts	Yes: assessments mostly at day 5, day 14	• Fmoc-FF/RGD hydrogel supported sustained viability and morphology. • Fmoc-FF/RGD had more cells with functional CYP450 compared to the 2D and Matrigel cultures.	No long-term culture beyond ~18 days; No passaging/expansion shown; Limited donor variety (n≈3) and no in vivo validation.	Fmoc-FF alone was insufficient to support cell survival or function.
Shoemaker et al., 2020 [34]	In vitro: PHHs, 3D organ-on-a-chip PerfusionPal insert system	Yes: functional readouts taken at days 4 and 7 post-plating	PerfusionPal + Blood Substitute significantly increased and prolonged CYP450 activity.	Short-term study (7 days)	Blood Substitute mimics hemoglobin oxygen delivery, improving in vitro relevance.
Sengupta et al., 2020 [32]	In vitro: PHHs co-cultured with MEFs	Yes	SHPCs maintained on MEFs retained differentiated morphology and metabolite-processing capacity through multiple passages.	Only acetaminophen metabolism assessed; Small sample size (n = 2).	Use of mouse feeder cells may complicate translational or xenogeneic concerns.
Gamboa et al., 2021 [35]	In vitro: PHHs, 3D liver organoid culture	Yes: (Multiple time points)	Optimized medium (EM + FSK + OSM) enhanced organoid expansion, and HNF4α and ALB expression	Adult donor-derived hepatocyte expansion remained less robust than fetal-derived or HepG2 organoids.	Freeze–thaw stability demonstrated over 2-week and 1-month post-thaw culture.
Biswas et al., 2022 [29]	In vitro: PRHs, 3D scaffolds	Yes: assessments mostly at day 5 and day 14.	• The IΔF + sLEM hybrid scaffold supported improved viability and enhanced albumin expression compared to collagen I controls. • Scaffold mimicked liver biomechanical environment and was proteolytically stable and biocompatible.	Comparison limited to collagen I; broader controls (e.g., Matrigel) not mentioned.	• Scaffold may be scalable and suitable for hepatocyte transplantation, drug testing, or regenerative models. • The dipeptide IΔF hydrogel was thoroughly characterized via TEM, CD spectroscopy, ThT staining, and rheological testing.
Mazari-Arrighi et al., 2022 [36]	In vitro: PRHs, 3D cell-fiber culture with 3T3-CM	Yes: monitored over 30 days in vitro	Hepatocytes in cell fibers + 3T3CM proliferated ~2.4-fold by day 4 and maintained ~46% viability through day 30. Albumin secretion, urea synthesis, CYP1A1 activity sustained through 30 days only in the 3T3CM condition.	Limited mechanistic insight into proliferation triggers	• Technology supports reproducibility across time due to fiber format.

Aflatoxin B_1_ (AFB_1_), Epithelial Cell Adhesion Molecule (EpCAM), Mitogen-activated Protein Kinase (MEK), Microphysiological Systems (MPS), Primary Rat Hepatocytes (PRH).

**Table 3:** Summary of studies that used transcriptional reinforcement and optimized isolation methods.

Authors.	Form of Modulation	Physical Conditions	Signaling Pathway Targeted	Number of Passages (TE or FM)	Findings	Limitations	Additional Notes
Fan et al., 2022 [37]	Genetic: Lentiviral overexpression of transcription factors Foxa3 and Hnf4a	In vitro: PRHs on collagen coated plates	FoxA3 and Hnf4α	20 passages **TE**	Overexpression of Foxa3 and Hnf4a enhances hepatocyte proliferation and maintains hepatic functions for up to 30 days in vitro	- No in vivo validation of functionality or safety - No RNA-seq or global transcriptomic profiling - Potential off-target effects of lentiviral integration not discussed	Cryopreserved cells retained morphology and growth
J Green et al. 2017 [38]	Mechanical and enzymatic two-stage hepatocytes isolation	Human Liver tissue of variable weights (7.8–600 g)	-	-	• Tissue weight ≥50 g was significantly associated with higher hepatocyte viability (>65%). • Average viability across all samples ~73 ± 13%; yield ~0.64 ± 0.19 × 10^6^ viable cells/g tissue. • No correlation between tissue steatosis or intracellular triglyceride and cell viability.	Only assessed short-term hepatocyte viability and basic function.	• Offers a protocol suitable for small, non-perfusable liver fragments. • Enables broader use of surgical surplus tissue for hepatocyte isolation.

## Data Availability

No new data were created or analyzed in this study. Data sharing is not applicable to this article.

## Abbreviations

The following abbreviations are used in this manuscript:

2D	Two-Dimensional
3D	Three-Dimensional
AFB1	Aflatoxin B_1_
AFP	Alpha-Fetoprotein
CYP450	Cytochrome P450
ECM	Extracellular Matrix
EGF	Epidermal Growth Factor
EGTA	Ethylene Glycol Tetraacetic Acid
EpCAM	Epithelial Cell Adhesion Molecule
FGF10	Fibroblast Growth Factor 10
FM	Functional Maintenance
HBV	Hepatitis B Virus
HDV	Hepatitis D Virus
HGF	Hepatocyte Growth Factor
HLCs	Hepatocyte-like Cells
HM	Hepatocyte Medium
HNF4A	Hepatocyte Nuclear Factor 4 Alpha
HPCs	Human Hepatic Progenitor Cells
iHPCs	Induced Hepatic Progenitor Cells
IL-6	Interleukin 6
iPSC	induced Pluripotent Stem Cell
LBDXL	Latrunculin B, Blebbistatin, Dasatinib, XAV939, LY294002
LDL	Low-Density Lipoprotein
MASLD	Metabolic Dysfunction-Associated Steatotic Liver Disease
MEFs	Mouse Embryonic Fibroblasts
MPS	Microphysiological Systems
NOG	NOD/SCID/IL2Rγnull
NPCs	Non-Parenchymal Cells
NSG	Nonobese Diabetic (NOD)-Cg-Prkdcscid Il2rgtm1Wjl/SzJ
OSM	Oncostatin M
OTC	Ornithine Transcarbamylase
PEG	Polyethylene Glycol
PHHs	Primary Human Hepatocytes
PHs	Primary Hepatocytes
PKA	Protein Kinase A
PLGA	Poly(lactic-co-glycolic acid)
PMHs	Primary Mouse Hepatocytes
PPHs	Primary Pig Hepatocytes
PRH	Primary Rat Hepatocytes
SHPCs	Small Hepatocyte Progenitor Cells
SMs	Small Molecules
TE	True Expansion
TGF-β	Transforming Growth Factor Beta
TK	Thymidine Kinase
TNFα	Tumor Necrosis Factor-α
VPA	Valproic Acid
YAP	Yes-associated Protein

## References

[1] Brolén G., Sivertsson L., Björquist P., Eriksson G., Ek M., Semb H., Johansson I., Andersson T.B., Ingelman-Sundberg M., Heins N. (2010). Hepatocyte-like cells derived from human embryonic stem cells specifically via definitive endoderm and a progenitor stage. J. Biotechnol..

[2] Kaur I., Vasudevan A., Rawal P., Tripathi D.M., Ramakrishna S., Kaur S., Sarin S.K. (2023). Primary Hepatocyte Isolation and Cultures: Technical Aspects, Challenges and Advancements. Bioengineering.

[3] Vilas-Boas V., Cooreman A., Gijbels E., Van Campenhout R., Gustafson E., Ballet S., Annaert P., Cogliati B., Vinken M. (2019). Primary hepatocytes and their cultures for the testing of drug-induced liver injury. Adv. Pharmacol..

[4] Zhang K., Zhang L., Liu W., Ma X., Cen J., Sun Z., Wang C., Feng S., Zhang Z., Yue L. (2018). In Vitro Expansion of Primary Human Hepatocytes with Efficient Liver Repopulation Capacity. Cell Stem Cell.

[5] Chen Y., Tang D., Wu H., Wu Y., Yuan T., Zhang H., Jiao Y., Yu W., Yan H. (2021). Assessment of long-term functional maintenance of primary human hepatocytes to predict drug-induced hepatoxicity in vitro. Arch. Toxicol..

[6] Dwyer B.J., Macmillan M.T., Brennan P.N., Forbes S.J. (2021). Cell therapy for advanced liver diseases: Repair or rebuild. J. Hepatol..

[7] Asrani S.K., Devarbhavi H., Eaton J., Kamath P.S. (2019). Burden of liver diseases in the world. J. Hepatol..

[8] Sun Z., Yuan X., Wu J., Wang C., Zhang K., Zhang L., Hui L. (2023). Hepatocyte transplantation: The progress and the challenges. Hepatol. Commun..

[9] Forbes S.J., Gupta S., Dhawan A. (2015). Cell therapy for liver disease: From liver transplantation to cell factory. J. Hepatol..

[10] Fu G.-B., Huang W.-J., Zeng M., Zhou X., Wu H.-P., Liu C.-C., Wu H., Weng J., Zhang H.-D., Cai Y.-C. (2018). Expansion and differentiation of human hepatocyte-derived liver progenitor-like cells and their use for the study of hepatotropic pathogens. Cell Res..

[11] Garnier D., Li R., Delbos F., Fourrier A., Collet C., Guguen-Guillouzo C., Chesné C., Nguyen T.H. (2018). Expansion of human primary hepatocytes in vitro through their amplification as liver progenitors in a 3D organoid system. Sci. Rep..

[12] Michalopoulos G.K. (2017). Hepatostat: Liver regeneration and normal liver tissue maintenance. Hepatology.

[13] Feng M., Kong R., Pan Y. (2019). The breakthrough in primary human hepatocytes in vitro expansion. Cancer Biol. Med..

[14] Gebhardt R., Matz-Soja M. (2014). Liver zonation: Novel aspects of its regulation and its impact on homeostasis. World J. Gastroenterol..

[15] Xie H., Li G., Fu Y., Jiang N., Yi S., Kong X., Shi J., Yin S., Peng J., Jiang Y. (2024). A two-step strategy to expand primary human hepatocytes in vitro with efficient metabolic and regenerative capacities. Stem Cell Res. Ther..

[16] Hu H., Gehart H., Artegiani B., Löpez-Iglesias C., Dekkers F., Basak O., Van Es J., Chuva de Sousa Lopes S.M., Begthel H., Korving J. (2018). Long-Term Expansion of Functional Mouse and Human Hepatocytes as 3D Organoids. Cell.

[17] Igarashi R., Oda M., Okada R., Yano T., Takahashi S., Pastuhov S., Matano M., Masuda N., Togasaki K., Ohta Y. (2025). Generation of human adult hepatocyte organoids with metabolic functions. Nature.

[18] Guo R., Jiang M., Wang G., Li B., Jia X., Ai Y., Chen S., Tang P., Liu A., Yuan Q. (2022). IL6 supports long-term expansion of hepatocytes in vitro. Nat. Commun..

[19] Jiang M., Guo R., Ai Y., Wang G., Tang P., Jia X., He B., Yuan Q., Xie X. (2023). Small molecule drugs promote repopulation of transplanted hepatocytes by stimulating cell dedifferentiation. JHEP Rep..

[20] Sun P., Zhang G., Su X., Jin C., Yu B., Yu X., Lv Z., Ma H., Zhang M., Wei W. (2019). Maintenance of Primary Hepatocyte Functions In Vitro by Inhibiting Mechanical Tension-Induced YAP Activation. Cell Rep..

[21] Li G., Zeng M., Yan Z., Cai S., Ma Y., Wang Y., Li S., Li Y., Zhong K., Xiao M. (2024). HDAC inhibitors support long-term expansion of porcine hepatocytes in vitro. Biomed. Pharmacother..

[22] Unzu C., Planet E., Brandenberg N., Fusil F., Cassano M., Perez-Vargas J., Friedli M., Cosset F., Lutolf M.P., Wildhaber B.E. (2019). Pharmacological Induction of a Progenitor State for the Efficient Expansion of Primary Human Hepatocytes. Hepatology.

[23] Hao J., Wang Z., Ren J., Cao S., Xie Z., Yang J., Li J., Ding W., Li J., Han Z. (2025). Single-cell multi-omics deciphers hepatocyte dedifferentiation and illuminates maintenance strategies. Cell Prolif..

[24] Vorrink S.U., Ullah S., Schmidt S., Nandania J., Velagapudi V., Beck O., Ingelman-Sundberg M., Lauschke V.M. (2017). Endogenous and xenobiotic metabolic stability of primary human hepatocytes in long-term 3D spheroid cultures revealed by a combination of targeted and untargeted metabolomics. FASEB J..

[25] Chang S.-Y., Voellinger J.L., Van Ness K.P., Chapron B., Shaffer R.M., Neumann T., White C.C., Kavanagh T.J., Kelly E.J., Eaton D.L. (2017). Characterization of rat or human hepatocytes cultured in microphysiological systems (MPS) to identify hepatotoxicity. Toxicol. Vitr..

[26] Bell C.C., Hendriks D.F.G., Moro S.M.L., Ellis E., Walsh J., Renblom A., Puigvert L.F., Dankers A.C.A., Jacobs F., Snoeys J. (2016). Characterization of primary human hepatocyte spheroids as a model system for drug-induced liver injury, liver function and disease. Sci. Rep..

[27] Bell C.C., A Dankers A.C., Lauschke V.M., Sison-Young R., Jenkins R., Rowe C., E Goldring C., Park K., Regan S.L., Walker T. (2018). Comparison of Hepatic 2D Sandwich Cultures and 3D Spheroids for Long-term Toxicity Applications: A Multicenter Study. Toxicol. Sci..

[28] Brown J.H., Das P., DiVito M.D., Ivancic D., Tan L.P., Wertheim J.A. (2018). Nanofibrous PLGA electrospun scaffolds modified with type I collagen influence hepatocyte function and support viability in vitro. Acta Biomater..

[29] Biswas S., Vasudevan A., Yadav N., Yadav S., Rawal P., Kaur I., Tripathi D.M., Kaur S., Chauhan V.S. (2022). Chemically Modified Dipeptide Based Hydrogel Supports Three-Dimensional Growth and Functions of Primary Hepatocytes. ACS Appl. Bio Mater..

[30] MacPherson D., Bram Y., Park J., Schwartz R.E. (2021). Peptide-based scaffolds for the culture and maintenance of primary human hepatocytes. Sci. Rep..

[31] Peng W.C., Logan C.Y., Fish M., Anbarchian T., Aguisanda F., Álvarez-Varela A., Wu P., Jin Y., Zhu J., Li B. (2018). Inflammatory Cytokine TNFα Promotes the Long-Term Expansion of Primary Hepatocytes in 3D Culture. Cell.

[32] Sengupta S., Johnson B., Seirup M., Ardalani H., Duffin B., Barrett-Wilt G.A., Stewart R., Thomson J.A. (2020). Co-culture with mouse embryonic fibroblasts improves maintenance of metabolic function of human small hepatocyte progenitor cells. Curr. Res. Toxicol..

[33] Rose S., Ezan F., Cuvellier M., Bruyère A., Legagneux V., Langouët S., Baffet G. (2021). Generation of proliferating human adult hepatocytes using optimized 3D culture conditions. Sci. Rep..

[34] Shoemaker J.T., Zhang W., Atlas S.I., Bryan R.A., Inman S.W., Vukasinovic J. (2020). A 3D Cell Culture Organ-on-a-Chip Platform With a Breathable Hemoglobin Analogue Augments and Extends Primary Human Hepatocyte Functions in vitro. Front. Mol. Biosci..

[35] Gamboa C.M., Wang Y., Xu H., Kalemba K., Wondisford F.E., Sabaawy H.E. (2021). Optimized 3D Culture of Hepatic Cells for Liver Organoid Metabolic Assays. Cells.

[36] Mazari-Arrighi E., Okitsu T., Teramae H., Aoyagi H., Kiyosawa M., Yano M., Chatelain F., Fuchs A., Takeuchi S. (2022). In vitro proliferation and long-term preservation of functional primary rat hepatocytes in cell fibers. Sci. Rep..

[37] Fan J.Y., Dama G., Liu Y.L., Guo W.Y., Lin J.T. (2023). Combinational Overexpression of Foxa3 and Hnf4a Enhance the Proliferation and Prolong the Functional Maintenance of Primary Hepatocytes. Mol. Biol..

[38] Green C.J., Charlton C.A., Wang L.-M., Silva M., Morten K.J., Hodson L. (2017). The isolation of primary hepatocytes from human tissue: Optimising the use of small non-encapsulated liver resection surplus. Cell Tissue Bank..

[39] Shulman M., Nahmias Y. (2012). Long-Term Culture and Coculture of Primary Rat and Human Hepatocytes. Epithelial Cell Culture Protocols.

[40] Nuciforo S., Heim M.H. (2021). Organoids to model liver disease. JHEP Rep..

[41] Afonso M.B., Marques V., van Mil S.W., Rodrigues C.M. (2023). Human liver organoids: From generation to applications. Hepatology.

[42] Kim Y., Kang M., Mamo M.G., Adisasmita M., Huch M., Choi D. (2025). Liver organoids: Current advances and future applications for hepatology. Clin. Mol. Hepatol..

